# What Does the Brain Have to Keep Working at Its Best? Resilience Mechanisms Such as Antioxidants and Brain/Cognitive Reserve for Counteracting Alzheimer’s Disease Degeneration

**DOI:** 10.3390/biology11050650

**Published:** 2022-04-24

**Authors:** Davide Maria Cammisuli, Ferdinando Franzoni, Giorgia Scarfò, Jonathan Fusi, Marco Gesi, Ubaldo Bonuccelli, Simona Daniele, Claudia Martini, Gianluca Castelnuovo

**Affiliations:** 1 Department of Psychology, Catholic University, 20123 Milan, Italy; davide.cammisuli1@unicatt.it; 2 Department of Clinical and Experimental Medicine, University of Pisa, 56126 Pisa, Italy; ferdinando.franzoni@unipi.it (F.F.); giorgiascarfo91@gmail.com (G.S.); jonathan.fusi@gmail.com (J.F.); ubaldo.bonuccelli@unipi.it (U.B.); 3 Department of Translational Research on New Technologies in Medicine and Surgery, University of Pisa, 56126 Pisa, Italy; marco.gesi@med.unipi.it; 4 Department of Pharmacy, University of Pisa, 56126 Pisa, Italy; simona.daniele@unipi.it (S.D.); claudia.martini@unipi.it (C.M.); 5 Psychology Research Laboratory, Istituto Auxologico Italiano IRCCS, 28824 Milan, Italy

**Keywords:** antioxidants, aging, Alzheimer’s disease, mild cognitive impairment, subjective cognitive decline, brain reserve, cognitive reserve, rehabilitation

## Abstract

**Simple Summary:**

Alzheimer’s disease currently represents one of the major challenges of modern society in relation to social and medical costs. As people age, they often experience mild changes in cognitive functioning that may be due to an initial degeneration of cerebral networks. Advances in neurobiology research including antioxidants intake and brain capacity to resist damage is relevant in order to support elderly people in the adoption of healthy lifestyles able to counteract dementia onset.

**Abstract:**

Here we performed a narrative review highlighting the effect of brain/cognitive reserve and natural/synthetic antioxidants in exerting a neuroprotective effect against cognitive deterioration during physiological and pathological aging. Particularly, we discussed pathogenesis of Alzheimer’s disease, brain and cognitive reserve as means of resilience towards deterioration, and evidence from the literature about antioxidants’ role in sustaining cognitive functioning in the preclinical phase of dementia. During aging, the effects of disease-related brain changes upon cognition are reduced in individuals with higher cognitive reserve, which might lose its potential with emerging cognitive symptoms in the transitional phase over the *continuum* normal aging-dementia (i.e., Mild Cognitive Impairment). Starting from this assumption, MCI should represent a potential target of intervention in which antioxidants effects may contribute—*in part*—to counteract a more severe brain deterioration (alongside to cognitive stimulation) causing a rightward shift in the trajectory of cognitive decline, leading patients to cross the threshold for clinical dementia later.

## 1. Introduction: Population Aging and Dementia Emergency

The average human lifespan is rapidly increasing and the maintenance of functional well-being in older age represents a current challenge in modern societies. The number of elderly people will dramatically increase in the near future as a consequence of progressive population aging. By 2050, 25% of people living in Europe and North America could be aged 65 or over [1]. Cognitive functioning is a major determinant of quality-of-life in the elderly and plays a critical role for the maintenance of personal and instrumental autonomy and functional abilities. Dementia currently represents a serious burden because of medical and social costs. The number of people with dementia worldwide is predicted to increase to 131.5 million by 2050 [2]. Alzheimer’s disease (AD) is the most common neurodegenerative disorder that causes dementia in the elderly.

Prevalence of AD rises continuously with age both in men and in women, doubling approximately every 5 years between the ages of 50 and 80 years and slowing in the oldest age groups [3]. About 2–3% of AD cases are due to autosomal dominant mutation, while the rest are sporadic cases and are prevalent in the aging population [4]. The onset of AD is insidious, since the underlying pathology is believed to be active for many years before the cognitive loss becomes evident; thus, strategies preventing deterioration in the elderly are therefore needed.

## 2. From Normal Aging to Alzheimer’s Disease

The aging brain undergoes many changes comprising biochemical, molecular, structural and functional ones that make individuals vulnerable to neurodegeneration. AD is a progressive neurodegenerative condition, for which advancing age represents a major risk factor [5]. For sporadic forms of AD, increasing age is recognized as the principal risk factor while the allele ɛ4 of the apolipoproteinE gene (i.e., apoE-ɛ4) represents the most important genetic risk factor for such a clinical condition [6]. Additionally, diabetes, obesity and other vascular illnesses are associated with dementia development [7]. The presence of extracellular senile plaques of insoluble β-amyloid peptide (Aβ) and neurofibrillary tangles composed of phosphorylated tau protein (P-tau) in the neuronal cytoplasm constitute the hallmarks of AD [8]. Amyloid plaques and neurofibrillary tangles are also observed in the aging human brain, even in people without dementia [2].

Although the mechanism of brain deterioration in AD is still debated, it is believed that it leads to atrophy and neuron death resulting from excitotoxicity processes, collapse in calcium homeostasis, inflammation and other factors [9]. As a result, brain networks sustaining episodic memory, learning and other relevant cognitive functions show a deficiency leading to a progressive cognitive impairment.

In AD, a long prodromal period exists. The characterization of Mild Cognitive Impairment (MCI) as a clinical entity different from AD for functional independence maintenance first allowed for the definition of a pattern of decline that is also divergent from normal aging, consisting of change in cognition over time (as reported by the patient, a reliable informant or a skilled clinician), and objective deficits on neurocognitive testing [10]. Further, the National Institute on Aging-Alzheimer′s Association (NIA-AA) criteria defined MCI as being *due to* AD “*as those symptomatic but not-demented individuals whose primarily underlying pathophysiology of AD*” [11]. MCI *due to* AD is characterized by the presence of memory impairment, a progressive decline in cognition over months to years and lack of evidence for vascular, traumatic or other medical causes of cognitive decline [11]. The likelihood of MCI progression to dementia has been estimated to occur at a rate 3 to 5 times higher than normal cognition, with an annual rate of 12% in the general population and 20% in populations at higher risk [12].

Moreover, the concept of Subjective Cognitive Decline (SCD) has been recently investigated as an earlier indicator of AD [13], with evidence suggesting that it may predict a faster conversion into MCI and dementia [14]. SCD is conceived as a self-experienced decline in cognitive functioning with regard to a previous normal cognitive status; individuals also report adequate performances on cognitive testing used to classify MCI [15]. With population aging, older adults’ concerns about cognitive decline constitute a relevant topic that frequently arises during medical examination. Most individuals notice some cognitive changes during aging. Specifically, population-based studies in older adults without cognitive impairment documented that from 50% to 80% of them report some forms of perceived decline when they are asked about it [16,17]. However, the majority of individuals with SCD do not decline towards dementia [15].

## 3. Brain and Cognitive Reserve as Resilience Mechanisms to Brain Deterioration

The concept of the ‘reserve’ accounts for individual differences in susceptibility to brain changes due to the aging process or AD-related pathology [18]. The common conceptualization of ‘reserve’ distinguishes an “hard” or neurological aspect from an “intellectual” or “functional” aspect. While *brain reserve* (BR) is based on the amount of available neural substrate (e.g., brain size, synapses density, dendritic branching), *cognitive reserve* (CR) is posed as a moderator between brain changes and clinical outcomes of elderly people resulting in coping with deterioration of the nervous system by using preexisting cognitive processes or compensatory strategies [19,20]. However, the current thinking is that reserve constitutes a dynamic aspect and a modifiable characteristic of the brain over the life span and that BR and CR are interdependent among them. This conception has led some authors to conclude that a clear separation between BR and CR is not a reflection of current biology [21]. Because of its theoretical construct, CR is usually inferred by proxy variables including measures of education and occupational attainment, intelligence, level of engagement in leisure or lifestyle activities, socioeconomical status and early life experiences [22].

A model of brain deterioration sustains the idea that when the number of functioning neurons or their connections falls below a critical level/threshold, individuals present with symptoms of cognitive impairment [23]. According to this conceptualization, individuals with high CR may reach a threshold for dementia diagnosis later than those with a lower resource [24]. They would hold an efficient set of neural networks or a wider repertoire of innate abilities or cognitive strategies enriched by environmental exposure leading them better compensate for loss more effectively [24]. Neuroimaging studies have further provided evidence that measures of CR are related to BR, including neural efficiency and capacity, brain volume and white matter integrity, neurotransmission and cerebrovascular health [22].

Some studies have further shown that among patients with MCI or dementia, the level of CR as measured by proxy variables modulates the relationship between neurocognitive abilities and clinical status/pathology, such as amyloid and tau, atrophy, brain metabolisms and cerebral perfusion [22]. BR and CR really act as moderators between neuropathology (e.g., brain atrophy) and its clinical manifestations (e.g., cognitive symptoms) since greater brain and cognitive reserve have been associated with beneficial outcomes, specifically a reduced risk of dementia [18]. 

## 4. Antioxidants as Resilience Mechanisms to Brain Degeneration

Interest in antioxidants’ capacity in contrasting dementia deterioration derived from the observation that oxidative stress may contribute to AD pathology [25]. In fact, the presence of extensive oxidative stress is a characteristic of the AD brain [26]. The redox imbalance in the AD brain is derived from a mitochondrial dysfunction and/or an altered homeostasis. Particularly, transition metals including copper (Cu), zinc (Zn) and iron (Fe) are crucial for synaptic plasticity and their impaired transport or accumulation cause free-radical genesis. This reactive oxygen species (ROS)-induced neurotoxicity is responsible for an increased β-amyloid production and aggregation and tau phosphorylation and polymerization [27]. In addition, the AD brain is characterized by activated microglia that usually reacts to β-amyloid deposition with production of inflammatory cytokines. In turn, the reactive microglia generates free-radical maintaining redox-imbalance, thus promoting neurodegenerative process [28].

Astrocytes, a type of glial cell, are also involved in brain pathology like AD [29]. Astrocytes play a relevant role for numerous brain functions, such as maintenance of neurotransmitter pools, immune surveillance, metabolism, synaptic formation and plasticity, and formation of the myelin sheath; in addition, they regulate vasodilation by controlling the nitric oxide pathway and protect neuronal function through their antioxidant activity [30,31]. The antioxidant activity of astrocytes is strictly dependent on their metabolism, and an interconnection between pentose–phosphate pathway and glutathione activity is recognized [32].

It is also well-known that the reduced form of glutathione (GSH) exerts an antioxidant effect through its conversion to the oxidized form of glutathione (GSSG). Subsequently, GSSG is converted back to GSH by nicotinamide adenine dinucleotide phosphate (NADPH). The use of NADPH produces NADP+, which is subsequently reduced to NADPH again by the pentose phosphate pathway. Thus, it seems that a link between the high activity of the pentose phosphate pathway in astrocytes and the antioxidant activity of astrocytes exists [31,32,33]. Furthermore, by releasing GSH into the extracellular environment, astrocytes exert an additional antioxidant action [34]. 

Melatonin (N-acetyl-5-methoxy-tryptamine) has been reported to have antioxidant effects, which are useful in preventing cognitive decline, too [35]. Melatonin is a ubiquitous hormone produced by the pineal gland and secreted into the bloodstream and it is involved into the regulation of the circadian rhythm, energy metabolism and mitochondrial biogenesis [36]. Furthermore, it has a free radical scavenging activity on neurons [37]. Melatonin reduces oxidative damage to the DNA, lipids and proteins and exerts its antioxidant activity as a direct scavenger by an up-regulation of antioxidant enzymes and a reduction in the activation of pro-oxidant enzymes [38]. Due to its chemical nature, it is also able to act against specific antioxidant species: peroxyl radical (LOO•), hydroperoxide (H_2_O_2_), hydroxyl radical (•OH), superoxide anion (•O_2−_), peroxynitrite derivatives (ONOO−) and singlet oxygen (1O_2_) [39]. Closely related to melatonin, the circadian rhythm, in turn, appears to play a pivotal role in control and production of oxidative species [40]. Beyond controlling the light-dark rhythm, *Bmal1*—known as the master clock gene, the deletion of which completely ablates all rhythmic activity throughout the organism—is specifically involved in controlling tissue homeostasis by directly regulating ROS levels. *Bmal1* deletion induces age-dependent astrocyte proliferation and microglia activation. This condition leads to an increase in ROS, resulting in neurodegeneration [41].

## 5. Additional Role of Nutrition and Physical Activity

A diet rich in antioxidants may reduce inflammation, which is associated with the risk of dementia [25]. Elderly people with higher intake of vitamin E and C either by diet or supplements have slower cognitive decline and lower risk of AD at old age [25]. Yasuno and colleagues [42] examined the effects of supplements daily intake consisting of antioxidants combination in a sample of elderly people (72.7 ± 4.8 yrs.) without dementia (i.e., n-3 polyunsaturated fatty acid, lycopene and ginkgo biloba extract). After controlling for contributing factors, the researchers found improvement in cognitive functions with a larger effect size for apoE-ɛ4 carriers at 3-year follow-up [26]. However, a recent meta-analysis [43] exploring the effect of antioxidants vitamins on cognitive functioning of non-demented older people indicated that only two studies specifically report some favorable effects upon cognitive functioning for β-carotene and for vitamin C intake in cognitively normal older adults. Specifically, in a large randomized controlled trial, namely ‘*Physician Health Study*’, a long-term supplementation (i.e., mean treatment duration, 18 years) with β-carotene (i.e., 50 mg on alternate days) in men older than 65 years was associated with improved global cognition [44]. Furthermore, vitamin C was not associated with cognitive changes over time in women of ≥65 years of age with cardiovascular disease, but showed a protective effect against new cardiovascular events [45]. According to Gillette-Guyonnet [46], select antioxidants including vitamins E, C, carotenes, polyphenols (i.e., flavonoids), and enzymatic cofactors of superoxide dismutase and glutathione peroxidase (i.e., zinc, selenium, manganese), may reduce neuronal damage and death from oxidative reactions by inhibiting the generation of the ROS, lipid peroxidation, apoptosis, protein oxidation, damage to cell membranes and/or DNA and beta-amyloid toxicity or deposition. However, a double-blind, placebo-controlled, parallel-group, randomized clinical trial involving a large cohort of patients with mild to moderate AD showed that 2000 IU/d of alpha tocopherol (Vitamin E) compared with placebo resulted in slower functional decline and reduced the caregiver’s burden but was unable to reduce the rate of MCI conversion into AD [47,48]. By contrast, Ito and colleagues [49] documented that the consumption of a composite supplement containing food-derived antioxidants, i.e., astaxanthin and sesamin of 6 mg/die and 10 mg/die, respectively, significantly improved psychomotor and processing speed in a sample of MCI patients. Astaxanthin, a red carotenoid found in salmon, shrimp, crab, and microalgae, is thought to alleviate oxidative stress-related brain dysfunctions [50]. Similar to astaxanthin, sesamin, a major lignan found in sesame extract, also produced neuroprotective effects and has been found to relieve the cerebral damage [51]. Furthermore, Twendee X (TwX) is a supplement containing a strong antioxidative mix of eight antioxidants, which has been shown to have a clinical and therapeutic benefit in AD model mice [52]. Although vitamins C and E were reported to have no therapeutic effect for dementia, a combination of different antioxidants has been shown to have a stronger effect than single antioxidant vitamins intake [53]. In fact, in a randomized controlled trial researchers evaluated the efficacy of TwX, a supplement containing a strong mix of eight antioxidants consisting of coenzyme Q10, niacin amid, L-cystine, ascorbic acid, succinic acid, fumaric acid, L-glutamine, and riboflavin. They reported an improvement in global cognition of MCI patients after a 6-month treatment in comparison to individuals assuming placebo [53].

By a systematic review of the literature, we also documented that a regular participation in moderate-intensity aerobic training (which typically implies exercise sufficient to elevate heart rate or Vo2 to approximately 60% of the maximum for 50/60 min 3 days a week) improves global cognitive status, logical memory, inhibitory control and divided attention in people with MCI [54]. Physical exercise brings improvements in cognitive functions accompanied by structural and functional changes of brain regions in MCI, especially as lower rates of brain atrophy and higher hippocampal efficiency [55]. In this regard, aerobic exercise might modify risk factors and pathological mechanisms associated with cognitive deterioration and concur in delaying dementia onset. 

**Figure 1:**
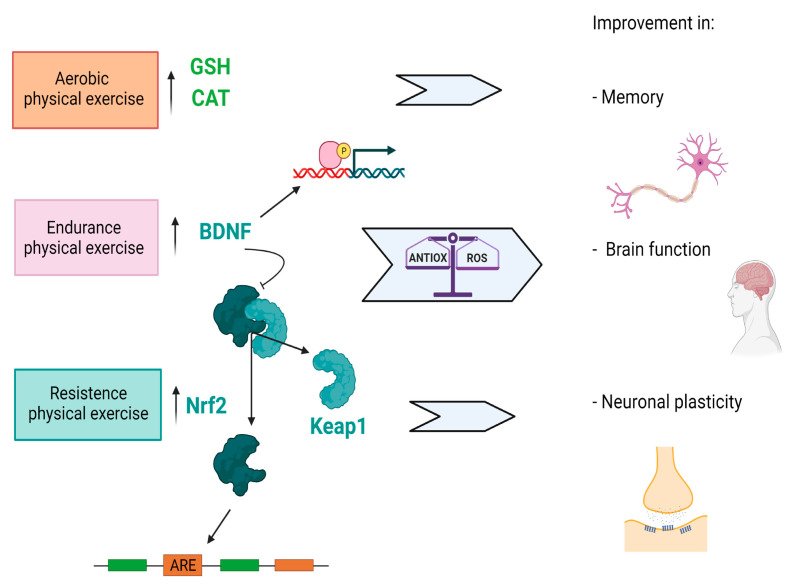
A representation of physical activity effects on brain functioning. *Notes*: The figure shows how aerobic, endurance and resistance physical exercise promote an upregulation of endogenous antioxidants, such as glutathione peroxidase (GHS), superoxide dismutase and catalase (CAT) and increases Brain-derived Neurotrophic Factor (BDNF) levels. This is responsible for the activation of nuclear factor erythroid 2-related factor 2 (Nrf2) which, once separated from Keap1, translocates into the nucleus and triggers promoter sequences called ‘ARE’. Such a process leads to a balance between the cellular antioxidant defences and the formation of free radicals (ROS) in order to obtain an improvement of cerebral performance and of synaptic neuroplasticity.

Regular physical exercise plays a crucial role in brain aging, since it leads to enhancement of neuroplasticity, increasing growth factor expression, decreasing inflammatory states and also acts as a buffer against oxidative stress [56]. Low level of ROS, which are produced intermittently for a short period of time during physical training protocols, activate intracellular signaling, positively affecting the brain to react against stress. Conversely, moderate levels of ROS generation over a long period or higher generation due to intensive exercise induce structural and functional damage [57]. Remarkably, both endurance and resistance physical exercise increase oxidative modification of proteins, nucleic acids and lipids. The main adaptive response to such kinds of exercise is related to the upregulation of endogenous antioxidants, such as glutathione peroxidase (GHS), superoxide dismutase and catalase (CAT) [58]. Finally, regular physical exercise increases levels of Brain-derived Neurotrophic Factor (BDNF) [59]. In particular, BDNF triggers the nuclear factor erythroid 2-related factor 2 (Nrf2), a cellular regulator of antioxidant defense system [60] that controls the expression of several enzymes to protect brain cells from oxidants, electrophiles and inflammatory agents [61] and to maintain mitochondrial function, cellular redox and protein homeostasis [62,63,64] (Figure 1).

## 6. Discussion: Antioxidants Capacity and Increased BR/CR

Normal aging is associated with deterioration of cognitive function and accumulation of neuropathological lesions occurring in AD. It represents the most common cause of dementia in elderly people and it is characterized by neurodegenerative alterations that progressively reduce cognitive and functional abilities of the patient. The biological course of AD is described as a 10/20-year preclinical phase, with a gradual accumulation of neuropathological lesions, in the absence of symptomatic cognitive impairment [65]. The duration of this period would also depend on the rate of the pathological progression offset by compensatory mechanisms usually referred as BR/CR [18]. We have documented the importance of enhancing the capacity of such reserve to prolong the pre-symptomatic phase of AD [25].

Cognitive decline may occur with marked variations among individuals. The abovementioned findings form research [19,20] reported that those individuals with greater BR better resist the effect of biological changes of dementia. It has been also suggested that the adequacy of nutrition before birth and in early formative years may have long-term consequences and shrinkage of the brain beginning in young adulthood implies that any insidious influence of diet will take place from that time onward [66]. Such a kind of observation leads to link a healthy nutrition to the brain efficiency over the life span. Moreover, beyond social engagement and individual’s factors playing a pivotal role in reserve capacity [67], a heathy lifestyle including a regular physical activity (especially aerobic exercise) in MCI patients is able to produce an improvement of cognitive functions through brain adaptations, by decreasing oxidizing species and neuroinflammation, as well as increasing antioxidants defense [54].

Cognitive decay can be influenced by a number of factors and the potential effect of antioxidants in human nutrition has become a topic of increasing scientific interest in the last few decades, since oxidative damage plays a critical role in the neuropathology of dementia. Starting from the fact that anti-oxidative capacity decreases with age, the prevention and treatment of cognitive impairment by specific antioxidants are welcome, especially in the preclinical phase of dementia. Remarkably, it has been documented that TwX is clinical beneficial for cognitive functions in MCI patients [53].

Findings from the literature highlight that the effects of age/disease-related brain changes upon cognition are reduced in individuals with higher BR/CR. Remarkably, in the Rush Memory and Aging Project, an ongoing prospective cohort study on aging and dementia, it has been specifically documented that high CR is associated with preserved global cognitive function, episodic memory and working memory, even in the presence of brain pathology, pointing out the key role of high CR accumulation in the prevention of cognitive decline [68]. A recent systematic review has also suggested that physical activity as a practice of a healthy lifestyle may contribute to CR and attenuate the damaging impact of brain changes upon cognition during time [69].

Education, occupation attainment, and leisure activities differently contribute to the reserve capacity. Specifically, CR increases during life through continuing education that has been found to be associated with brain volumes in non-demented elderly people [70]. Similarly, people with demanding occupations in their life have continuously stimulated divergent thinking and mental flexibility as executive functions allowing them to better adapt to different contexts. Remarkably, stimulating jobs descrease the risk of dementia in old age, as reported by a recent multichohort study [71]. Additionally, a prospective 21-year study demonstrates a significant association between higher level of participation in leisure activities at baseline and decrease risk of dementia of AD type [72]. In fact, motivating settings and engagement in recreational and social activities may improve the autonomy of patients in daily living. These considerations have led researchers to evaluate the clinical manifestations of dementia in relation to the individual differences, environmental and social factors beyond the neurodegenerative process, increasing the importance of staying mentally and socially active during aging.

**Figure 2:**
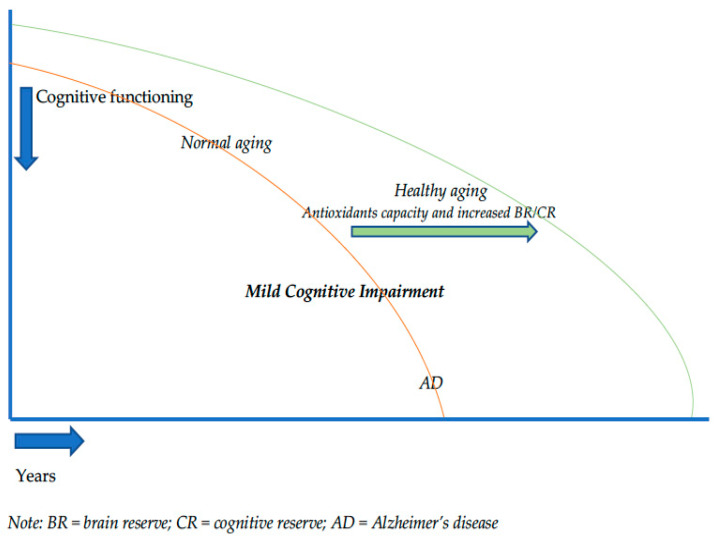
Trajectory of cognitive decline and resilience mechanisms.

Further, there is some evidence suggesting that both natural and synthetic antioxidants may improve cognition in people with MCI that might be thought as a consequence of an initial failure of BR/CR because of emerging cognitive symptoms not present in normal aging. This would indicate benefits of class-specific natural/synthetic antioxidants (i.e., astaxanthin and sesamin, *Twendee X*) intake during MCI, as a target condition characterizing the transitional phase between physiological and pathological aging, in order to rightward shift the trajectory of cognitive decline, thus crossing the threshold for clinical dementia later (Figure 2).

## 7. Conclusions: Depicting the Trajectory of Cognitive Decline

In the light of preliminary evidence, MCI should be considered a critical period for antioxidant intervention. However, larger randomized controlled trials are necessary in the future to confirm the therapeutic role of class-specific antioxidants by investigations comparing potential effects of interventions in different study arms (i.e., SCD, MCI and mild AD). We also suggest that future rehabilitation protocols in the early phase of cognitive impairment should adopt a combined intervention of strong antioxidants therapy associated with physical activity (and cognitive stimulation) in order to slow down or postpone MCI conversion into AD. Education, occupational attainment and engagement in leisure activities enhance reserve capacity and may alter the effects of neurodegeneration. Finally, the efficacy of other natural products with antioxidant properties such as luteolin, quercetin, apigenin that have been shown to have positive effects in initial studies [73] should be studied in depth also in MCI research to find out their possible therapeutic effects.

## Data Availability

Not applicable.
